# The Impacts of Gender and Subject on Experience of Competence and Autonomy in STEM

**DOI:** 10.3389/fpsyg.2019.01432

**Published:** 2019-06-27

**Authors:** Sabrina Sobieraj, Nicole C. Krämer

**Affiliations:** Social Psychology: Media and Communication, University of Duisburg-Essen, Duisburg, Germany

**Keywords:** gender, STEM – science technology engineering mathematics, competence, autonomy, motivation

## Abstract

In most societies, women are less likely to choose a science, technology, engineering and mathematics (STEM)-related study program than men. This problem persists despite numerous initiatives aimed at fostering the uptake of STEM subjects by women, who represent an underutilized source of talent in a time of great need for STEM professionals. Many reasons for women’s avoidance of the path into STEM-related areas have been discussed, including weaker mathematical skills, implicit gender stereotypes or structural deficits in school education. One variable which is presumably at the core of decisions regarding a specific study subject is motivation. We aim to look in greater depth at the basis for motivation by referring to self-determination theory (SDT). Here, we specifically focus on the needs for *competence* and *autonomy* which represent pivotal sources of motivation, effective performance and psychological well-being and are assumed to be positively correlated with academic achievement and perseverance. In line with previous SDT research, we assume that self-perceptions during STEM studies contribute to experiences of competence and autonomy and may be responsible for gender disparities. To examine whether and how a sex-specific perception of autonomy and competence influences decisions regarding STEM subjects, we conducted a survey study of Master’s students (*N* = 888; 461 female, 427 male), who were enrolled either in STEM or non-STEM subjects, and asked about students’ motivations, perceived competence (e.g., self-efficacy) and autonomy (e.g., volitional decision for a study major). The results revealed several main effects of study major and only a small number of interaction effects of sex and subject. For example, non-STEM students were more likely to enroll due to their stronger interest in their subject, signifying higher autonomy, while STEM students were more likely to select their subject according to their families’ wishes. The comparison between female and male STEM students revealed that males perceived more self-efficacy and reported more leadership aspirations while female STEM students have lower perceptions of their own competence, especially regarding perceived future competences.

## Introduction

Despite economists’ repeated calls for more professionals in science, technology, engineering and mathematics (STEM) in both the short and medium term, the number of students deciding to enroll in STEM still does not meet the economic needs (Dasgupta and Stout, 2014). Moreover, Dasgupta and Stout (2014) report that women are still underrepresented in STEM, resulting in an underutilized source of talent. Indeed, even if women do start an academic career in science or engineering, they do not go on to achieve the highest positions: In the academic sector in the United States, only 21% of full professors in science and only 5% of full professors in engineering are female (Shen, 2013). This is a long standing problem, with Wickware (1997) having acknowledged over 20 years ago that women leave research more often than men.

Many reasons have been discussed regarding why women avoid the path into STEM, such as weaker mathematical skills, structural deficits in school education, and gender stereotypes (see Mavriplis et al., 2010; Smeding, 2012; Wang and Degol, 2013). Gender stereotypes are probably one of the most intensively debated of the proposed reasons. For example, it has been found that the picture of a scientist is more congruent with the stereotype of a man than of a woman (e.g., Carli et al., 2016). This stereotype has often been found among adults, and even more impressively, it has also been demonstrated in children: Using the draw-a-scientist method, in which children are instructed to draw a scientist, children consistently draw Caucasian males (e.g., Finson, 2002; Buldu, 2006; Losh et al., 2008; Miller et al., 2018). Such findings reflect how at least scientists or engineers (e.g., Fralick et al., 2009) are perceived. Based on this, it is conceivable that the implicit knowledge about STEM professionals and gender stereotypes influences students’ decisions for or against STEM. For instance, women might be less motivated to decide in favor of STEM because according to stereotypes, they are expected to be less talented and interested in STEM professions.

One seminal theory which strives to explain human motivation is Self-Determination Theory (SDT). The theory states that there are external drivers/motivations (e.g., monetary incentives) and internal drivers/motivations (e.g., personal interests) for human behavior, and that internal motivations in particular can lead to persistence in goal achievement, such as graduating in a STEM major. SDT assumes that the motivation to engage in a specific behavior is dependent on whether this behavior is perceived to satisfy the needs for *competence, autonomy* and *relatedness.* The satisfaction of these basic psychological needs leads to high levels of intrinsic motivation, effective performance and well-being (Ryan and Deci, 2014). In this research, we suggest that specifically the perceptions of competence and autonomy are relevant for the selection of a study subject and might contribute to decisions for or against STEM. We do not focus on relatedness as we were more interested in the rather “self-centered” needs of competence and autonomy as potential drivers of gender differences regarding choice of study. Particularly if there are gender differences in perceived competence and autonomy in the context of study-related self-perceptions (operationalized as self-efficacy, self-esteem, leadership orientation and causality orientation regarding competence and autonomy), and in study interest and reasons for choosing a study subject, this may help to detect possible reasons for the gender gap in STEM. In the present study, we assess these constructs in students with STEM subjects in contrast to non-STEM subjects, with a special focus on differences between female and male undergraduates. By choosing a broad approach including motivations, interests as well as autonomy- and competence-related self-perceptions, we hope to gain further insights into the reasons why women are underrepresented in STEM.

### Stereotype Reasons

Stereotypes about women and men exist in all cultures (e.g., Prentice and Carranza, 2002; Cuddy et al., 2008). In Western cultures, they include the assumption or expectation that women have a warm nature, are caring, gentle, and friendly, and act in a communal manner, while men are expected to be competitive, competent, goal-oriented, and mathematically skilled, and to act agentically (e.g., Cuddy et al., 2008). Importantly, individuals strive to fit into their gender role, because violating existing norms can have detrimental consequences such as discrimination and harm (e.g., Burgess and Borgida, 1999). In turn, this can have reinforcing effects on the individual who behaves unconventionally and is penalized, or behaves gender-congruently and is rewarded or at least not penalized. Possible effects of this may be that individuals adapt their behavior and/or change their self-related cognitions to fit into their gender roles.

The stereotype of a scientist is suggested to encompass many of the attributes that are also associated with stereotypical beliefs about males (Carli et al., 2016; Hand et al., 2017; Ramsey, 2017), such as being more agentic and less communal. However, Carli et al. (2016) note that the higher the proportion of female scientists in a particular field, the more the stereotype in that field mirrors stereotypical beliefs about women.

A meta-analysis of five decades of draw-a-scientist studies revealed that the scientist stereotype seems to evolve in childhood (elementary school, middle school) and increasingly strengthens with age (high school; Miller et al., 2018). The authors attributed this to children’s/teenager’s observation from their environment (e.g., textbooks, extracurricular experiences) in which female scientists are still underrepresented. This interpretation is supported by findings that male scientists are more often represented in TV formats than female scientists (e.g., Long et al., 2010; Steinke and Tavarez, 2018). Similar stereotypical beliefs apply for STEM professionals in general and have been demonstrated in high-school students and their teachers (e.g., Hand et al., 2017), college students (e.g., Piatek-Jimenez et al., 2018) and even among STEM professionals themselves (e.g., Farrell and McHugh, 2017). Notably, while most investigated groups show an implicit pro-male STEM bias, female STEM professionals demonstrate a slight pro-female gender bias (Farrell and McHugh, 2017). Nevertheless, the majority of findings point in favor of men.

Given that stereotypes, such as gender stereotypes, can affect actual perception and behavior (Bem, 1993; Master et al., 2017), it is easily conceivable that stereotypes about scientists can also influence women’s and men’s behavior, for example when they decide whether or not to engage in science, technology, engineering and math. Master and Meltzoff (2016) argued that such stereotypes can act as barriers preventing girls from studying STEM subjects. Based on this assumption, they conducted two experimental studies in which they varied the classroom environment, making stereotypes about computer scientists either salient (e.g., Star Trek posters) or not (e.g., nature posters). The authors found that boys’ interest in computer science was not affected by the classroom design, but girls’ interest was affected: The girls were three times more interested in computer science when they were in the non-stereotypical compared to the stereotypical environment. This is in line with findings regarding the “stereotype threat,” which claims that stigmatized groups are threatened by stereotypes and perform less effectively when stereotypes are primed. For example, Spencer et al. (1999) found that women performed worse in solving a mathematical problem when they were made aware of the stereotypical belief that females are less mathematically skilled than men. In addition, Steffens et al. (2010) demonstrated that the knowledge about STEM stereotypes (here, math-gender stereotype) can become influential from the age of nine. The authors found that this knowledge affects academic achievements, enrolment preferences and the academic self-concept of girls but not of boys.

There is a reasonable amount of available knowledge concerning the stereotypes about STEM professionals and concerning which structural features can influence the decision for STEM study subjects, for example sufficient financial coverage or 12th-grade math achievements (Wang, 2013). However, less is known about the self-perceptions of STEM professionals or those who wish to become STEM professionals (STEM students). Moreover, women and men in STEM may perceive their environment, such as the campus climate, differently (Gayles and Ampaw, 2014), which can hinder their graduation. Wang and Degol (2013) stated that individuals make rational choices based on their abilities in order to maximize their outcomes relative to their costs, and suggested that these individual expectations to succeed influence their decisions regarding STEM or any other academic path. In sum, individuals’ introspection concerning their motivation, abilities and capabilities might be a decisive factor influencing women’s and men’s paths into STEM professions. An influential theory which specifically focuses on motivations in order to be able to explain people’s behaviors and choices is SDT which was therefore chosen as a theoretical framework to specifically target people’s competence and autonomy.

#### Self-Determination Theory

Deci and Ryan (2011) proposed SDT as a macro theory consisting of six different mini-theories, such as *cognitive evaluation theory*, which focuses on the increase in intrinsic motivation, or *basic psychological needs theory*, which represents the core of SDT (for an overview see Deci and Ryan, 2011). Deci and Ryan (2000) described the needs for *competence, autonomy* and *relatedness*, which can be satisfied or thwarted by social contexts (Ryan and Deci, 2014). These needs are pivotal sources of motivation, effective performance and psychological well-being. We assume that the perceptions of specifically *competence* and *autonomy* as more “self-centered” needs may serve as valuable sources for making decisions in favor of or against STEM and that they might develop differentially in women and men.

*Autonomy* refers to the feeling of volitional self-regulation of behavior (Legate and Ryan, 2014): “Autonomy concerns acting from interest and integrated values. When autonomous, individuals experience their behavior as an expression of the self, such that, even when actions are influenced by outside sources, the actors concur with those influences, feeling both initiative and value with regard to them.” (Ryan and Deci, 2002, p. 8). Furthermore, autonomy has been shown to positively contribute to interest in a subject (Black and Deci, 2000).

*Competence* refers to the experience of capability and effectiveness to achieve desired goals (Ryan and Deci, 2014). It “…leads people to seek challenges […] and to persistently attempt to maintain and enhance those skills and capacities through activity. Competence is not, then, an attained skill or capability, but rather is a felt sense of confidence and effectance in action.” (Ryan and Deci, 2002, p.7). According to Feltman and Elliot (2014), the perception of competence influences achievement outcomes, which can have an approaching character (success) or an avoiding character (failure). Striving for an approaching outcome can lead to creativity, optimal performance attainment and persistent interest. Avoiding outcomes, by contrast, lead to the opposite and to a higher probability of seeking easy rather than difficult goals/challenges. Jang et al. (2009) revealed that perceived competence is positively associated with academic achievements, and Wang and Degol (2013) summarized that girls who are confronted with STEM tasks are particularly vulnerable to perceiving themselves as lacking capability or as less competent.

Steele and Aronson (2005) stated that competence has a fragile nature, because a person who is confident in one task is not necessarily confident in another task (e.g., being good at art but a poor athlete). This fragility also reveals itself in the impact of stereotype threat (e.g., Spencer et al., 1999; Shapiro and Williams, 2012), as it shows that a specific competence (e.g., mathematical skills) can be shaken by mere priming.

One factor that shapes the perception of competence and autonomy refers to how individuals attribute the results of different actions. Deci and Ryan (1985) distinguished three types of causality orientations, which are uniquely represented in each individual: *autonomy orientation*, *controlled orientation* and *impersonal orientation*. Autonomy orientation refers to an internal attribution style in which the individual feels that he/she is in charge of his/her actions and consequences. Controlled orientation refers to an external attribution style in which environmental cues are held accountable for actions and outcomes. Impersonal orientation refers to the feeling that actions and consequences are beyond the individual’s control, and is associated with anxiety regarding incompetence (Ryan and Deci, 2014). High ratings in autonomy orientation are positively correlated with professional satisfaction and academic achievements (Stipek and Weisz, 1981; Findley and Cooper, 1983; Ng et al., 2006); controlled orientation is associated with rigidity and lower levels of wellness (Ryan and Deci, 2014) and shows a small negative correlation with academic performance (Lane et al., 2004). Both concepts are measured in the present study in order to assess the individual perception of competence and autonomy.

Research has shown that women tend to possess an attribution style in which especially success is attributed to external cues, while men tend to have an internal attribution style in which especially success is attributed to oneself (e.g., ability; Burgner and Hewstone, 1993). However, the attribution style depends on the domain: “with boys citing more competency in traditionally masculine activities (sports and math) and girls citing more competency beliefs in traditionally feminine activities (reading and music…).” (Mezulis et al., 2004, p. 714). Given that STEM is assumed to reflect a male domain, it is conceivable that based on gender stereotypes and STEM stereotypes, women in STEM possess a rather self-derogating attribution style (e.g., low autonomy orientation), in contrast to their male counterparts. Early research on female engineering majors revealed that one reason for dropping out may be that female students tend to attribute their successes to external causes rather than to their own capabilities (Nauta et al., 1999).

### Competence and Autonomy Perception

Another variable which is classically related to gender differences in male-dominated fields such as STEM (Zeldin et al., 2008) is self-efficacy, which contributes to the perception of competence. Bandura (1982) defined self-efficacy as the individual’s belief about her/his ability to solve a task or reach a goal. It determines whether or not an individual wishes to engage in an activity and the degree of effort and perseverance the individual invests. Furthermore, self-efficacy is predictive of academic development in terms of academic aspirations, performance and persistence (Multon et al., 1991; Bandura, 1993; Lane et al., 2004; Zajacova et al., 2005). Focusing on self-efficacy in mathematical problem solving, Pajares and Miller (1994) revealed that self-efficacy beliefs are more important than, for instance, prior experience with mathematics. In a longitudinal study, Marra et al. (2009) found that women with high self-efficacy ratings are more often willing to persist in male-dominated fields such as STEM. Moreover, Zeldin et al. (2008) found that male STEM professionals gain their self-efficacy beliefs from prior mastery experience and previous successes, while women rely on vicarious experiences (e.g., observing similar others in performing) and verbal persuasion (e.g., positive encouragement from others).

While self-efficacy is a broad concept, academic self-efficacy refers to goal achievement in the academic context and captures the individual’s specific beliefs about his/her confidence in solving academic tasks (Bong and Skaalvik, 2003). Similar to self-efficacy, academic self-efficacy is positively correlated with, for instance, academic performance and coping behavior (Chemers et al., 2001; Robbins et al., 2004; Gore, 2006). Zeldin et al. (2008) found that girls indicated having lower levels of science self-efficacy, which can predict lower interest in science. In a longitudinal study of STEM undergraduates who are underrepresented in STEM education and professions, MacPhee et al. (2013) reported that at the time of admission, women perceived themselves to have lower academic skills than their male counterparts, even though they did not show a weaker performance. By the time of graduation, however, women’s academic self-concept was equal to that of men – at least for the specific sample that was enrolled in a mentoring program. Referring to previous findings and stereotype research, it can be assumed that women, in contrast to men, lack self-efficacy beliefs (general and academic) and that this in turn may lead to a decreased desire to enter a STEM study program and less confidence in one’s success as a STEM professional.

Self-esteem represents another concept which can contribute to perceptions of competence and particularly to autonomy, because it – unlike self-efficacy - does not directly refer to capabilities. Self-esteem more broadly describes personal attitudes concerning one’s self-worth (Rosenberg et al., 1995). While Lane et al. (2004) revealed a positive correlation between self-esteem and academic success, other authors, such as Baumeister et al. (2003), reported that self-esteem is not a good predictor of academic success but does sometimes predict job performance. However, these authors acknowledged that high self-esteem scores can facilitate further engagement after failure.

A further variable which is – albeit more loosely - associated with one’s own perception of competence are leadership aspirations (Bass and Stogdill, 1990). As described by Ryan and Deci (2002) the perception of competence leads people to seek challenges. With regard to the challenge of aspiring leadership positions in STEM fields, however, there seems to be a distinct difference between men and women. The general lack of women in leadership positions (Amon, 2017) is even more pronounced in STEM fields (McCullough, 2011). On the one hand, this is caused by the lower number of women in STEM *per se* (McCullough, 2011), and on the other hand, perceived stereotypes and gender roles might also play a role. Amon (2017) found that female STEM graduates and postdoctoral researchers perceive various barriers concerning leadership in STEM, especially regarding the role conflict between being a woman and being in a leadership position, which is perceived as challenging. In particular, the effort that is perceived to be required for role transitioning (Amon, 2017) might lead women to balance costs and benefits, leading to fewer leadership aspirations than in men.

In conclusion, stereotype research suggests that stereotypes about STEM professionals resemble stereotypes about men and stand broadly in contrast to beliefs about women. These beliefs might be crucial, especially for women, when making decisions for or against STEM professions, for which choosing STEM subjects at university/college represents an important step. We draw on SDT as a theory on basic psychological needs which are described as pivotal sources of motivation and effective performance. We therefore measure the orientation specifically toward the here relevant “self-centered” needs of autonomy and competence by scales developed by Ryan and Deci (2014) and additionally assess the related constructs of self-efficiency, self-esteem and leadership orientation. Most importantly, we directly assess students’ perception of their motivation to choose their major and their study motivation. Adding to self-determination-theory measures, we rely on measures stemming from expectancy-value models of motivation (Kosovich et al., 2015). Also, as it was shown that decision making for educational trajectories represent complex processes (Becker and Hecken, 2009), that comprise, besides interests and abilities, motives for status maintenance (e.g., that families expect their children to follow their occupation), we added these aspects to classical motivation scales.

In a first step, we test whether there are general differences between STEM and non-STEM students. Therefore, we pose the following research question:

RQ1: How do STEM students (in contrast to non-STEM students) perceive their competence and autonomy? How do they perceive their self-efficacy, self-esteem and leadership aspiration and what study motivation and motivation for choosing their major do they report?

Moreover, research indicates that women in STEM face gender-STEM stereotypes, which can result, for instance, in stereotype threats or potential role conflicts (Amon, 2017), potentially affecting women’s self-perceptions and well-being. In view of such negative outcomes, it seems reasonable to ask whether female STEM students differ not only from female non-STEM students but also from male STEM and male non-STEM students – potentially showing greater motivation, competence and autonomy than the other groups. We therefore ask:

RQ2: How do female STEM students differ from female non-STEM students, male STEM students and male non-STEM students concerning their perceived competence and autonomy? How do they perceive their self-efficacy, self-esteem and leadership aspiration and what study motivation and motivation for choosing their major do they report compared to the other groups?

In order to scrutinize gender differences specifically within the group of STEM students, we ask the following research question:

RQ3: How do female and male STEM students differ in their perceived competence and autonomy? How do they differ regarding their self-efficacy, self-esteem and leadership aspiration and do they report different study motivations and motivations for choosing their major?

## Materials and Methods

### Procedure

To address our research questions, we conducted a survey study among Master’s students of the University of Duisburg-Essen, Germany. Approximately 43.001 students are enrolled (48% women; retrieved from uni-due.de, 2019) in at least one study program at the university, which ranks number eight in German universities concerning its number of students. Approximately 9600 master students were enrolled in all disciplines at the moment of conduct (2017). The disciplinary canon of faculties of the university comprises: humanities, social sciences, educational sciences, economic sciences, business administration, mathematics, physics, chemistry, biology, engineering, and medicine.

According to national guidelines no ethics vote was required for the present survey; however, the study adheres to ethical standards, which are made transparent through the following descriptions. Participants were fully debriefed about the purpose of the study and participants did not face any consequences if they canceled the survey. However, since the study was issued as an official survey by the university it was presented, discussed and approved by the rectorate of the University and the data protection officer. The study was part of a larger survey study focusing on the careers of young academics, which also included post-docs and Ph.D. candidates (these two groups are not focused on in the current paper). The students were invited to take part in an online survey to gather data on their study motivation, their perceptions of their own competence and autonomy, as well as sociodemographic characteristics (more details below). At the beginning of the survey participants were informed about the aim of the survey (to collect data about individual perceptions of their study programs and their career aspirations) and that their data will be saved anonymously. By clicking a check box they gave informed consent that they are of age and permit to capture their data, afterward they got access to the rest of the survey. Those not accepting these requirements were free to quit the survey without any consequences. After completing the survey participants were fully debriefed about the focus on gender differences. We did not inform about this in advance in order to prevent priming of gender stereotypical beliefs, which could have influenced responses. Participants did not receive any immediate payment but had the opportunity to take part in a prize draw. The potential winnings included minor amounts of money (e.g., 10 Euros up to 50 Euros, in total 500 Euros) and the chance to win a book allowance worth 1.000 Euros (split into 2 × 500 Euros). Students needed approximately 30 min to complete the survey.

### Measures

#### Sociodemographic Characteristics

The survey began with questions concerning students’ sociodemographic characteristics such as sex (biological category), nationality, and age. Furthermore, we asked about their school career, professional career, and academic career as well as of that their parents. All categories are based on the German school, academic and professional system and can only be partially compared to other countries’ systems. In cases in which no international equivalent exists we will present translations. The participants could only choose one category at a time.

Firstly, participants were asked at which type of school they received their university entrance exams/level (e.g., “Gymnasium [secondary school], “Gesamtschule [comprehensive school],” “received in a foreign country”). Afterward they were asked “where did you receive your first university degree (e.g., Bachelor’s degree)?” and could respond by the choices “at the University of Duisburg-Essen,” “at another university in Germany” and “at another foreign university.” In addition, we assessed in which field of study they received their first university degree, for instance, “Humanities,” “Engineering,” “Law Studies.” Finally, we asked for their final grade (“≤1.4; 1.5 – 1.9; 2.0 – 2.4; 2.5 – 2.9; 3.0 – 3.4; 3.5 – 4.0”; the lower the number, the better the grade with 1,0 representing the best grade and 4.0 representing the worst pass grade in the German tertiary education).

Concerning the educational and professional trajectories of participants’ parents we asked for the following choices within the school trajectory sections: “mittlere Reife” [graduating from a medium-track school], “Fachhochschulreife oder Allg. Hochschulreife oder Ähnliches [graduading from a higher-track school/qualification for university entrance or something comparable],” “no graduation,” “something else,” “I don’t know.” The categories of the academic education comprised: “university degree,” “doctorate”; “no academic degree,” “something else,” “I don’t know.” The categories in the professional career are “abgeschlossene Berufsausbildung [completed vocational training],” “weiterführende berufsqualifizierende Ausbildungsgänge [advanced vocational trainings],” “no completed vocational training,” “something else” and “I don’t know.”

#### Reasons for Choosing Majors

We asked for specific reasons (eight items) why students made the decision for their majors, including intrinsic (e.g., “my subject comes easily to me”) and extrinsic orientations (e.g., “I can make a lot of money”) and reasons, which stem from educational studies and seem to be decisive for study program choice alike (e.g., Ramseier, 2006; Kretschmann et al., 2017). In addition, we created ad-hoc items to assess motives for status maintenance (Becker and Hecken, 2009) and the autonomy of the decision. With this, we aimed to potentially show (1) a broader range of reasons for participants’ decisions and (2) to examine whether there are differences depending on gender and major. These items are such as “I chose my major because my family wanted me to” or “I chose my major because I did not get a place on my preferred study program” (for all items, means and standard deviation see Table 1). Items were rated on a 5-point rating scale (1 = not at all; 5 = absolutely). Due to their heterogeneity, for further analysis we consider the single items; no factors were formed.

**TABLE 1:** Descriptive statistics of reasons for study subject.

**Reasons**	***M***	***SD***	***N***
I know exactly what I will do as a professional after graduation	2.32	1.12	888
It reflects my interests	3.57	0.63	886
My subjects comes easily to me	3.25	0.70	887
I did not know what else to study	1.74	0.94	888
I can make a lot of money after graduation	2.26	0.98	887
I will have a lot of professional opportunities after graduation	2.82	0.91	886
My family wanted me to choose this major	1.37	0.72	888
I did not get a place on my preferred study program	1.42	0.82	888

#### Study Interest

To indicate their specific interest in their subject, students completed the Fragebogen zum Studieninteresse [Study Interest Questionnaire] by Schiefele et al. (1993). This short scale consists of nine items (α = 0.727), such as “If I had enough time, I would be more concerned with certain issues of my studies, beyond the exams” or “Even before my studies, the subject had a special significance for me.” Items were rated on a 4-point rating scale (0 = not at all; 3 = absolutely).

#### Study Motivation

To gather data on study motivation in terms of expectancy, value, and cost of study, we adapted the Expectancy-Value-Cost (EVC) scale by Kosovich et al. (2015) to refer to students’ study programs. Each of the three dimensions was measured with two items. For example, expectancy (α = 0.695) was measured with the statement “I believe that I can be successful in my study program,” value (α = 0.861) was measured with “I value my major,” and costs of study (α = 0.667) was measured with “I have to give up too much to do well in my major.” Items were rated on a 4-point scale (1 = strongly disagree; 4 = strongly agree).

#### Causality Orientation

To examine participants’ causality orientation, we used three selected situational vignettes by Deci and Ryan (1985, retrieved from selfdeterminationtheory.org, 2017), which are oriented toward achievement situations. Participants were asked to imagine a fictitious situation from day-to-day life and to make suggestions about their feelings and thoughts. For example: “You had a job interview several weeks ago. In the mail you received a form letter which states that the position has been filled. It is likely that you might think:…”, with the three responses “It’s not what you know, but who you know” (controlled orientation); “I’m probably not good enough for the job.” (impersonal orientation) and “Somehow they didn’t see my qualifications as matching their needs” (autonomy orientation). Participants had to make a choice on each statement on 7-point scales (1 = very unlikely that the participants would respond in this way; 4 = moderately likely; 7 = very likely), which represented one of three causal dimensions. Afterward the scores for the respective statements were added into these subscales (controlled orientation, α = 0.280; autonomy orientation, α = 0.360; impersonal orientation, α = 0.506) resulting in ratings from 3 (low manifestation within this orientation) to 21 (high manifestation within this orientation).

#### Self-Efficacy

General self-efficacy was measured with 10 items from the Skala zur Allgemeinen Selbstwirksamkeitserwartung [Generalized Self-Efficacy Scale] by Jerusalem and Schwarzer (1999), such as “I feel comfortable with difficulties because I can always rely on my abilities.” Items were rated on a 4-point rating scale (1 = disagree; 4 = agree). Scores were summed up into one scale (α = 0.812) ranging from 10 (low self-efficacy) to 40 (high self-efficacy).

We captured academic self-efficacy by using the BWS Skala [Occupational Self-Efficacy Scale] by Abele et al. (2000); we adapted the scale to refer to academia. The scale focuses on how to solve requirements and difficulties related to study, such as “I do not know if I really have the skills to study.” Items were rated on a 5-point rating scale (1 = not at all; 5 = absolutely; α = 0.765).

#### Self-Esteem

Self-esteem was measured using the 10-item scale by Collani and Herzberg (2003), a German version of the Rosenberg Self-Esteem Scale. Items comprise statements such as “On the whole, I am satisfied with myself”; statements were rated on a 4-point rating scale (1 = strongly disagree; 4 = strongly agree). Scores of items were added into a sum score resulting in a scale ranging from 10 (low self-esteem) to 40 (high self-esteem; α = 0.864).

#### Leadership Aspirations

Leadership aspirations were captured by asking students the question (Powell and Butterfield, 2013) of which positions they would wish to hold in future: top management, middle management, lower management or employee without a leadership position.

### Sample

The final sample comprised 888 Master’s students (461 female, 427 male), who would be graduating either in STEM (physics, chemistry, biology, engineering, mathematics) or non-STEM (humanities, social sciences, educational sciences) subjects. We excluded nine participants because they refrained from indicating their gender or categorized themselves as transsexual/intersexual/queer. A further three were excluded due to insufficient data quality (less than 60 percent of questions answered).

Students ranged in age from 20 to over 49; the majority were 25–29 years old (57.3%), followed by the group of 20–24 year-olds (30.3%). Eighty percent indicated that they were German nationals, while the remaining 20% came, for instance, from India, Turkey, or European countries. Students graduated (Bachelor’s degree or something comparable) from different disciplines: humanities (*n* = 190), social sciences (*n* = 148), art, music, design (*n* = 6), economic science (*n* = 26), natural science (*n* = 150), law studies (*n* = 1), engineering (*n* = 355), others (8). Four persons refrained from answering this question.

With regard to students’ family background, the largest proportion of students’ mothers had graduated from a medium-track school of the German three-tier school system (48.6%), followed by a higher-track school, corresponding to university entrance level (42.3%). Most of the mothers did not hold a university degree (65.3%), while approximately one third were university graduates (29.1%). More than half of the mothers had received vocational training (54.4%). By contrast, the largest proportion of students’ fathers had graduated from a higher-track school (50.1%), followed by a medium-track school (40%). Approximately half of the fathers did not have an academic degree (51.6%), while 42.3% were university graduates and 42.4% had received vocational training.

## Results

Before testing the research questions, we checked for assumptions (e.g., normally distributed data and moderate correlations of dependent variables, see Field, 2013) to run appropriate analyses. To examine RQ1-RQ3, we conducted MANOVAs, ANOVAs as well as Chi^2^ tests; detailed descriptions of tests and results will be consecutively reported. We used the whole sample (*N* = 888) for testing RQ1 and RQ2, while for testing RQ3 we refer to a smaller sample consisting of STEM students (*n* = 529). We will sequentially report results from RQ1 to RQ3.

### Effects of Subject

#### Effects of Subject on Reasons for Choosing Study Subjects

Regarding reasons for choosing their study subjects, we conducted 2 × 2 ANOVAs with subject (STEM vs. non-STEM) and gender (female, male) as independent variables and reasons as dependent variables (see Table 2), because assumptions for MANOVA testing were not fully met (e.g., low or no correlations between variables). Moreover, for dependent variables which did not meet assumptions for ANOVA testing we conducted Chi^2^ tests.

**TABLE 2:** Effects of subjects, gender and subject^*^gender on reasons for choosing study subject.

**Reasons**	***df***	***F***	***p***	**η^2^**
My subject comes easily to me				
Subject	1,883	19.86	<0.001	0.022
Gender		0.00	0.948	<0.001
Subject × Gender		1.03	0.310	0.001
I can make a lot of money after graduation				
Subject	1,883	135.78	<0.001	0.133
Gender		4.52	0.034	0.005
Subject × Gender		1.28	0.259	0.001
I will have a lot of professional opportunities after graduation				
Subject	1,882	88.46	<0.001	0.091
Gender		2.51	0.114	0.003
Subject × Gender		4.97	0.026	0.006
I know exactly what I will do as a professional after graduation				
Subject	1,884	0.39	0.530	<0.001
Gender		1.33	0.250	0.001
Subject × Gender		2.46	0.117	0.003

The single ANOVA referring to the statement that “their subject comes easily to them” revealed that non-STEM students agreed slightly stronger [*M* = 3.39; *SD* = 0.65; Levene’s test *F*(3,883) = 0.43, *p* = 0.730, for all variance terms see Table 2] with the statement than the STEM students (*M* = 3.16; *SD* = 0.72).

The ANOVA concerning the statement “I can make a lot of money after graduation” is significant (see Table 2). Although the Levene’s test is significant, *F*(3,883) = 6.30, *p* < 0.001, we adhere to the analysis because the ANOVA is fairly robust to violation of homogeneity (Bortz and Schuster, 2010; Field, 2013). STEM students agreed with the statement more often than non-STEM students (*M_STEM_* = 2.60; *SD* = 0.92; *M*_non–STEM_ = 1.76; *SD* = 0.82).

In addition, STEM students agreed more often to the reasoning that they will have a lot of professional opportunities after graduation [*M_STEM_* = 3.09; *SD* = 0.85; *M*_non–STEM_ = 2.44; *SD* = 0.86; Levene’s test *F*(3,882) = 3.86, *p* = 0.009]. No significant difference was found for the item “I know exactly what I will do as a professional after graduation” [Levene’s test *F*(3,884) = 11.08, *p* < 0.001].

Before conducting Chi^2^ tests for the remaining reasons we checked for distributions of answers in each cell (2 × 5) resulting from answers in 5 response categories (1 = not at all; 5 = absolutely) and two subjects (STEM vs. non-STEM). Noteworthy, distributions to the remaining reasons are left skewered and no answers in category 5 (absolutely) were provided resulting in a maximum of 2 × 4 cells. In cases in which cell counts were below 5 we collapsed the respective category with the next adjacent category, e.g., when response counts in category 4 were below 5 we collapsed it with category 3.

The Chi^2^ test is not significant, *χ^2^*(3) = 5.81, *p* = 0.121, φ = 0.08, for the reason “I did not get a place on my preferred study program” indicating that STEM and non-STEM students do not differ in their agreement to this statement.

Referring to the reason that the study subject reflects participants’ interest we merged categories 1 and 2, because of counts less than 5 in category 1. The Chi^2^ test was significant, χ^2^(2) = 8.81, *p* = 0.012, φ = 0.100. There is no difference between STEM (6.1%) and non-STEM (4.5%) students in choosing category 2; indicating that there is no difference depending on student’s major concerning low agreement to the statement. However, STEM students more often decided for category 3 (35.0%_STEM_; 26.8%_non–STEM_) indicating that they more often agreed moderately to the statement that they chose their major due to their interest. By contrast, non-STEM students decided more often for a stronger agreement (category 4) to the reason than STEM students (58.9%_STEM_; 68.7%_non–STEM_) indicating that non-STEM students rather chose their study subject based on their interests than STEM students.

Concerning the reason “I did not know what else to study” the test, χ^2^(3) = 14.47, *p* = 0.002, φ = 0.13, revealed that non-STEM students (60.5%) significantly decided more often for category 1, indicating more disagreement with this reason, than STEM students (50.9%).

Moreover, STEM students more often chose category 2 (23.6%_STEM_; 21.7%_non–STEM_) and category 4 (7.9%_STEM_; 2.8%_non–STEM_), while no significant difference for category 3 can be observed (17.6%_STEM_; 15.0%_non–STEM_). These results point in the direction that STEM students agree more often to the statement that they chose their study program because they did not know what else to study than their non-STEM counterparts.

For the reason “my family wanted me to study the subject” we merged category 4 with category 3 because of zero counts in single cells of category 4. The analysis revealed a significant Chi^2^ test, χ^2^(2) = 45.15, *p* < 0.001, φ = 0.23. STEM students significantly less often chose category 1 (66.5%_STEM_; 85.8%_non–STEM_) but more often category 2 (19.8%_STEM_; 10.9%_non–STEM_) and category 3 (13.6%_STEM_; 3.3%_non–STEM_). This pattern indicates that STEM students agreed more often that they chose their study subject because their families wanted them to than their non-STEM counterparts.

#### Effects of Subject on Study Interest

Regarding study interest, we conducted a 2 × 2 ANOVA that revealed that non-STEM students (*M*_non–STEM_ = 3.15; *SD* = 0.47) indicated more interest than did STEM students [*M_STEM_* = 3.02; *SD* = 0.45; Levene’s test *F*(3,872) = 0.45, *p* = 0.719, variance terms see Table 2].

#### Effects of Subject on Study Motivation

To test for potential differences between STEM and non-STEM students concerning their study motivation we checked for assumptions to conduct a MANOVA with the scales three sub-dimensions (*value*, *expectancy*, and *cost*), however, these were not fully met (e.g., partly low correlations; no normal distributions of residuals). Therefore, we conducted Chi^2^ tests for *expectancy* and *value* and an ANOVA for *cost*. For *expectancy* as well as *value* (2 = not at all, 8 = absolutely, resulting in categories 2–8) we had to collapse categories 2 and 3 (each below 5 counts) with category 4 to reach a critical number of counts. The test was not significant for *expectancy*, χ^2^(4) = 5.04, *p* = 0.283, φ = 0.08 indicating that STEM students and non-STEM students do not differ in their expectancies that they can be successful in their study program.

By contrast, the Chi^2^ test for *value* χ^2^(4) = 18.72, *p* < 0.001,φ = 0.15 is significant. STEM students less often chose category 4 (1.5%) compared to non-STEM students (2.8%), in contrast STEM students more often decided for category 8 (37.5%) than non-STEM students (21.4%). Students did not differ by subject concerning the remaining categories (category 5: 2.0%_STEM_, 2.1%_non–STEM_; category 6: 11.5%_STEM_, 9.7%_non–STEM_; category 7: 7.1%_STEM_, 4.2%_non–STEM_). This pattern of results indicates that STEM students value their study program more than their non-STEM counterparts.

For the subdimension of *cost* we run an ANOVA resulting in a significant effect [Levene’s test *F*(3,882) = 1.61, *p* = 0.186; variance terms see Table 2]. Means show that STEM students attribute more costs (*M* = 5.55, *SD* = 1.58) than non-STEM students (*M* = 4.78, *SD* = 1.45).

#### Effects of Subject on Causality Orientation

In respect to causality orientation we conducted a 2 × 2 MANOVA with the *control* and *impersonal orientation* as dependent variables after checking for assumptions. Since *autonomy* is not correlated to both other dimensions, we conducted a separate 2 × 2 ANOVA for this dimension.

The MANOVA revealed no significant differences concerning subject [Box’s test *F*(9,827532) = 8.65, *p* = 0.474; variance terms see Table 2] for *impersonal* and *control orientations*, as neither did the ANOVA for the *autonomy orientation* [Levene’s test *F*(3,879) = 0.54, *p* = 0.658; variance terms see Table 2]. This indicates that STEM and non-STEM students do not differ in the way they attribute the results of different actions.

#### Effects of Subject on Self-Esteem

Moreover, we conducted a 2 × 2 ANOVA with *self-esteem* as dependent variable. STEM students showed lower self-esteem ratings (*M_STEM_ =* 31.87; *SD =* 5.62) than did non-STEM students [*M*_non–STEM_ = 32.78; *SD* = 5.55; Levene’s test *F*(3,868) = 1.33, *p* = 0.268, for variance terms see Table 3].

**TABLE 3:** Effects of subjects, gender and subject^*^gender on study interest, study motivation, causality orientation, self-efficacy, academic self-efficacy, and self-esteem.

**Construct**	***df***	***F***	***p***	**η^2^**
Study interest				
Subject	1,872	13.44	<0.001	0.015
Gender		0.00	0.999	0.000
Subject × Gender		0.81	0.369	0.001
Study motivation				
Cost				
Subject	1,882	49.44	<0.001	0.053
Gender		0.01	0.938	<0.001
Subject × Gender		11.71	0.001	0.013
Causality orientation				
Autonomy				
Subject	1,879	0.95	0.329	0.001
Gender		10.73	<0.001	0.012
Subject × Gender		2.62	0.106	0.003
Controlled				
Subject	1,877	3.61	0.058	0.004
Gender		22.03	<0.001	0.025
Subject × Gender		0.85	0.357	0.001
Impersonal				
Subject	1,877	0.27	0.602	<0.001
Gender		35.04	<0.001	0.038
Subject × Gender		0.23	0.633	<0.001
Self-efficacy				
Self-efficacy				
Subject	1,855	0.18	0.669	<0.001
Gender		14.85	<0.001	0.017
Subject × Gender		0.13	0.669	<0.001
Academic self-efficacy				
Subject	1,855	3.45	0.064	0.004
Gender		2.65	0.104	0.003
Subject × Gender		1.14	0.285	0.001
Self-esteem				
Subject	1,868	7.28	0.007	0.008
Gender		1.09	0.297	0.001
Subject × Gender		2.36	0.125	0.003

#### Effects of Subject on (Academic) Self-Efficacy

In addition, we run a 2 × 2 MANOVA with self-efficacy and academic self-efficacy [Box’s test *F*(9,813227) = 8.12, *p* = 0.57], which resulted in insignificant effects of subject for both subdimensions (for variance terms see Table 3). STEM and non-STEM students seem not to differ concerning their perceived self-efficacy and academic self-efficacy.

#### Effects of Subject on Leadership Aspirations

Finally, we conducted a Chi^2^ test to examine whether STEM students differ from non-STEM students regarding their leadership aspirations (4 response categories). The test yielded a significant difference, χ^2^(3) = 24.93, *p* < 0.001, φ = 0.17: STEM and non-STEM students did not differ regarding striving for positions as employees without leadership duties (8.9%_STEM_; 7.5%_non–STEM_) and lower-management positions (15.7%_STEM_; 19.3%_non–STEM_). However, non-STEM students were found to strive significantly more often for positions in middle management (59.5%_non–STEM_; 48.2%_STEM_), while STEM students strive significantly more often for top management positions (27.1%_STEM_; 13.7%_non–STEM_). In sum, therefore, STEM students aspire higher leadership positions than non-STEM students.

### Interaction Effects of Subject × Gender

#### Interaction Effects of Subject × Gender on Reasons for Choosing Study Subjects

With regard to RQ2 (interactions of subject and gender), the ANOVAs revealed one significant interaction effect for the reason “I will have a lot of professional opportunities after graduation” (variance term see Table 2), indicating that male STEM students (*M_STEM_* = 3.18; *SD* = 0.79) see the most opportunities after graduation, even more than their female counterparts (*M_STEM_* = 2.92; *SD* = 0.94), while there was no difference in the lower ratings of female non-STEM students (*M*_non–STEM_ = 2.45; *SD* = 0.88) and male non-STEM students *(M*_non–STEM_ = 2.40; *SD* = 0.78). The other interaction effects were not significant (see Table 2).

Moreover, the Chi^2^ tests revealed three effects for subject and gender. Ratings were given from 1 = “not at all” to 5 = “absolutely,” mirroring in 5 response categories. Owing the fact that no answers were provided in category 5 we downsized response categories to a maximum of 4 categories.

For the reason “It reflects my interests” we merged response categories 1–3 due to lower counts than 5 in single cells, resulting in the two response categories: 3 and 4. The analysis revealed a significant effect, χ^2^(3) = 11.98, *p* = 0.007, φ = 0.12. Male STEM students (43%) more often chose category 3 than female non-STEM students (29.5%). By contrast, female non-STEM students (70.5%) select more often category 4 than male STEM students (57%). No other differences emerged (category 3: 37.6%_female  STEM,_ 37.6%_male  non–STEM;_ category 4: 62.4%_female  STEM,_ 62.7%_male  non–STEM_). This response pattern indicates that the groups of female non-STEM students and male STEM students significantly differ from each other, while the other groups share some similarities. Female non-STEM students seem to choose their major more often because it reflects their interests, while male STEM students more often rather disagree with this reason.

To conduct the analysis for the reason “I did not get a place on my preferred study subject” we had to merge categories 4 through 2 because of low cell counts in categories 3 and 4, resulting in two response categories: 2 and 1. The test is significant, χ^2^(3) = 13.52, *p* = 0.004, φ = 0.12. Male non-STEM students chose more often category 1 (86.9%) than their male STEM counterparts (68.8%), while it is opposite for category 2 (13.1%_male  non–STEM_; 31.2%_male  STEM_). No further differences for the other categories emerged (category 1 = 76%_female  non–STEM_; 76.9%_female  STEM_; category 2: 24%_female  non–STEM_; 23.1%_female  STEM_). It seems that male STEM students agree slightly more to the reason that they chose their study program because they did not get a place on their preferred study program than their male non-STEM counterparts, while no difference concerning the female groups can be observed.

We collapsed categories 3 and 4, resulting in 3 response categories (see Table 4) for the item “My family wanted me to,” to run the Chi^2^ test. The analysis revealed a significant effect, χ^2^(6) = 55.17, *p* < 0.001, φ = 0.18. Referring to the column’s proportions (see Table 4) female non-STEM students chose category 1 more often than female STEM students and male STEM students. In addition, the male non-STEM students more often disagree with this reason than the male STEM students. These results indicate that the groups of female and male non-STEM students more often disagree with the statement that they chose their study program because their families wanted them to than male STEM students. Within category 2 the only difference occurred between male STEM students and female non-STEM students, indicating that male STEM students chose this category more often. Concerning category 3 male STEM students are more often in this category compared to female non-STEM students. In addition, female STEM students chose this category more often than female non-STEM students. This pattern of results indicates that predominantly male STEM students, but also female STEM students chose their study program because their families wanted them to in contrast to the group of female non-STEM students.

**TABLE 4:** Crosstable for the reason “My family wanted me to.”

			**Female**	**Female**	**Male**	**Male**
**Category**		****	**non-STEM**	**STEM**	**non-STEM**	**STEM**
1,00	Count		241_a_	137_b,c_	67_a,c_	215_b_
	Expected count		204.4	138.2	62.4	254.9
	% within		87.6%	73.7%	79.8%	62.7%
	2,00 Count		27_a_	30_a, b_	12_a, b_	75_b_
	Expected count		44.6	30.2	13.6	55.6
	% within		9.8%	16.1%	14.3%	21.9%
3,00	Count		7_a_	19_b_	5_a, b_	53_b_
	Expected count		26.0	17.6	7.9	32.4
	% within		2.5%	10.2%	6.0%	15.5%

Each subscript letter denotes a subset of Gender^*^Subject categories whose column proportions do not differ significantly from each other at the 0.05 level.

Moreover, the analysis of the statement “I did not know what else to study” was not significant, χ^2^(6) = 10.69, *p* = 0.099, φ = 0.08, indicating that none of the groups differed in the agreement with this statement.

#### Interaction Effects of Subject × Gender on Study Motivation

The ANOVA concerning the subdimension of *costs* within the study motivation construct revealed an interaction effect for subject and gender (variance term see Table 3). The means indicated that men in STEM scored highest regarding expected costs (*M =* 5.68; *SD =* 1.54), followed by women in STEM (*M =* 5.30; *SD =* 1.54), women in non-STEM (*M* = 4.88; *SD* = 1.45) and men in non-STEM (*M =* 4.47; *SD =* 1.42).

Moreover, we analyzed the interaction of subject and gender for the *value* subdimension after collapsing categories 2 through 5 (2 = not at all, 8 = absolutely), resulting in the response categories 5–8. The Chi^2^ test is significant, χ^2^(9) = 23.70, *p* = 0.005, φ = 0.09. Referring to the column’s proportions female and male non-STEM students more often decide for category 5 than female STEM students and male STEM students, indicating that non-STEM students more often agree on a lower moderate level concerning study program value.

Moreover, in category 6 the only difference emerged for male STEM students and male non-STEM students indicating that male non-STEM students are more often in this category. Within category 7 no difference between groups proportions occurred.

For category 8 the analysis revealed that female STEM students chose this answer more often than female non-STEM students and male non-STEM students. In addition, male STEM students are more often in this category than male non-STEM students (for proportions see Table 5). No difference emerged between female and male STEM students. This pattern of results indicates that STEM students, especially female STEM students differ from the non-STEM groups in the sense that they value their study subject more.

**TABLE 5:** Crosstable for the subdimension value of study motivation scale.

		**Female**	**Female**	**Male**	**Male**
**Category**		**non-STEM**	**STEM**	**non-STEM**	**STEM**
5,00	Count	33_a_	8_b_	12_a_	23_b_
	Expected count	23.6	16.0	7.1	29.3
	% within	12.0%	4.3%	14.5%	6.7%
6,00	Count	62_a, b_	40_a, b_	24_b_	62_a_
	Expected count	58.4	39.5	17.6	72.6
	% within	22.5%	21.5%	28.9%	18.1%
7,00	Count	28_a_	17_a_	9_a_	46_a_
	Expected count	31.0	21.0	9.4	38.6
	% within	10.2%	9.1%	10.8%	13.5%
8,00	Count	152_a, b_	121_c_	38_b_	211_a, c_
	Expected count	162.0	109.6	48.9	201.5
	% within	55.3%	65.1%	45.8%	61.7%

Each subscript letter denotes a subset of Gender^*^Subject categories whose column proportions do not differ significantly from each other at the 0.05 level.

The analysis for the subdimension of *expectancy* is not significant, χ^2^(6) = 9.04, *p* = 0.171, φ = 0.07, indicating that the groups do not differ in their expected study success.

#### Interaction Effects of Subject × Gender on Study Interest, Causality Orientation, Self-Esteem, and Self-Efficacy

The interaction effects of gender and subject are not significant for the (sub-) dimensions of study interest, causality orientation, self-esteem and self-efficacy (for variance terms see Table 2), indicating that the groups cannot be distinguished by these variables.

### Gender Differences Within STEM Students

To examine RQ3, we compared female and male STEM students referring to the same methods as for RQ1 and RQ2.

#### Effects of STEM Student’s Gender on Reasons for Choosing Study Subject

The analyses identified several differences between female and male STEM students regarding the reasons why they chose their STEM subjects. Concerning the reason “My subject comes easily to me” the ANOVA revealed no significant effect, *F*(1,527) = 0.60, *p* = 0.440, η^2^ = 0.001 [Levene’s test *F*(1,526) = 0.45, *p* = 0.833], indicating that female and male STEM students do not differ in this regards.

By contrast, the ANOVA for “I can make a lot of money” is significant, *F*(1,526) = 7.01, *p* = 0.008, η^2^ = 0.013 [Levene’s test *F*(1,526) = 6.94, *p* = 0.009] indicating that male STEM students (*M_male_* = 2.68; *SD* = 0.87) agreed slightly more often than female STEM students (*M*_female_ = 2.46; *SD* = 0.99).

The same pattern of responses emerged for “I will have lot of opportunities after graduation,” *F*(1,525) = 7.56, *p* = 0.001, η^2^ = 0.020 [Levene’s test *F*(1,525) = 3.16, *p* = 0.076]. Male STEM students (*M_male_* = 3.18; *SD* = 0.79) agree slightly stronger than female STEM students (*M*_female_ = 2.92; *SD* = 0.04).

The ANOVA for the item “I know exactly what I will do professionally after graduation” points in the same direction, *F*(1,527) = 6.10, *p* = 0.014, η^2^ = 0.011 [Levene’s test *F*(1,527) = 0.36, *p* = 0.546] that male STEM students (*M_male_* = 2.39; *SD* = 1.02) agreed slightly more than their female counterparts (*M*_female_ = 2.16; *SD* = 1.08).

After collapsing categories 1 and 2, resulting in response categories 2–4, of the item “it reflects my interests,” due to low cell counts, the Chi^2^ test was not significant, χ^2^(2) = 1.44, *p* = 0.487, φ = 0.05, indicating that women and men in STEM do not differ in their response behavior.

Moreover, none of the remaining reason analyses were significant: “My family wanted me to,” χ^2^(3) = 6.77, *p* = 0.080, φ = 0.11, “I did not know what else to study,” χ^2^(3) = 0.25, *p* = 0.968, φ = 0.02, and “I did not get a place on my preferred study program,” χ^2^(3) = 4.04, *p* = 0.258, φ = 0.09, indicating that male and female STEM students cannot be discriminated by these variables.

#### Effects of STEM Student’s Gender on Study Interest

The ANOVA focusing on study interest as dependent variable was not significant, *F*(1,524) = 0.61, *p* = 0.436, η^2^ = 0.001 [Levene’s test *F*(1,524) = 0.31, *p* = 0.861], indicating that female and male STEM students show the same interest in their study program.

#### Effects of STEM Student’s Gender on Study Motivation

We run an ANOVA with the subdimension *cost* as dependent variable, which revealed a significant effect, *F*(1,526) = 7.69, *p* = 0.006, η^2^ = 0.014 [Levene’s test *F*(1,527) = 0.06, *p* = 0.815] indicating that male STEM students (*M* = 5.30, *SD* = 1.54) score higher on this scale than female STEM students (*M* = 5.68; *SD* = 1.54).

For the Chi^2^ tests of *expectancy* and *value* we collapsed categories 2 through 5, resulting in response categories 5–8, due to low cell counts. The analyses revealed no significant effects, neither for *expectancy*, χ^2^(2) = 0.12, *p* = 0.941, φ = 0.02, nor for *value*, χ^2^(3) = 4.01, *p* = 0.260, φ = 0.09, indicating that female and male STEM students have similar expectancies toward their study success and the value of their study program.

#### Effects of STEM Student’s Gender on Causality Orientation

The ANOVA concerning the subdimension *autonomy* is not significant, *F*(1,523) = 2.00, *p* = 0.157, η^2^ = 0.004 [Levene’s test *F*(1,523) = 0.16, *p* = 0.693] indicating that female and male STEM students do not differ in the way they attribute results of actions to themselves.

Moreover, the MANOVA with its subdimensions *impersonal* and *control* [Box’s test *F*(3,4157256) = 5.08, *p* = 0.168] revealed significant effects for both dimensions [impersonal: *F*(1,523) = 30.23, *p* < 0.001, η^2^ = 0.055; control: *F*(1,523) = 22.33, *p* < 0.001, η^2^ = 0.041]. The analysis showed that female STEM students score higher (*M* = 16.23; *SD* = 2.80) on *control orientation* than male STEM students (*M* = 14.95; *SD* = 3.05) indicating that female STEM students assume environmental cues more accountable for their actions and outcomes than male STEM students. The same response pattern occurred for the *impersonal orientation* (*M_female_* = 12.45; *SD* = 3.80; *M_male_* = 10.62; *SD* = 3.56), indicating that female STEM students have an increased feeling that actions and consequences are beyond their control in contrast with male STEM students.

#### Effects of STEM Student’s Gender on Self-Esteem

The analysis referring to STEM student’s self-esteem was not significant, *F*(1,520) = 0.17, *p* = 0.687, η^2^ < 0.001 [Levene’s test *F*(1,520) = 2.80, *p* = 0.095]. These result suggest that male and female STEM students do not differ in their self-esteem perception.

#### Effects of STEM Student’s Gender on Self-Efficacy

The MANOVA concerning self-efficacy and academic self-efficacy [Box’s test *F*(3,3828798) = 6.14, *p* = 0.107] revealed a significant effect for self-efficacy, *F*(1,508) = 12.76, *p* < 0.001, η^2^ = 0.025, showing that male STEM students (*M* = 29.54; *SD* = 4.21) score higher on the scale than their female counterparts (*M* = 28.10; *SD* = 4.61). There is no significant effect for academic self-efficacy, *F*(1,508) = 0.23, *p* = 0.631, η^2^ < 0.001, indicating no difference between female and male STEM students in this variable.

#### Effects of STEM Student’s Gender on Leadership Orientation

Finally, the Chi^2^ test revealed a significant difference in the leadership aspirations of female and male STEM students, χ^2^(3) = 42.16, *p* < 0.001,φ = 0.28. The frequencies show that female and male STEM students do not differ in striving for positions as employees without leadership duties (10.8%_females_; 7.9%_males_). However, female STEM students more frequently strive for positions within lower management (21.6%_females_; 12.6%_males_) and middle management (57.3%_females_; 43.3%_males_), while male STEM students more often strive for top management positions (36.3%_males_; 10.3%_females_) indicating higher leadership aspiration for males.

## Discussion

The current study focused on self-perceptions of STEM and non-STEM students with a special focus on female STEM students in terms of their study motivation, competence and autonomy. SDT suggests that confidence in one’s own abilities and capabilities (competence) and acting volitionally (autonomy) can determine an individual’s interest in a subject and academic achievements. The results of our survey study revealed that STEM and non-STEM students differ in their motivation, perceived competence and autonomy. A first indication of the different perceptions of STEM and non-STEM students emerged concerning their motivation.

First, STEM students scored lower than their non-STEM counterparts on general interest in their majors. In line with Black and Deci (2000), this lower interest might indicate that STEM students’ need for autonomy is met to a lesser degree compared to non-STEM students. A consideration of the reasons for choosing a study subject supports this assumption: STEM students did not choose their subjects based on their general interest; instead, their choice was determined more by a lack of ideas about what else they should study, because their family wished for them to choose STEM more often.

Second, the analyses showed that STEM students valued their subjects to a greater degree than did non-STEM students, and third, they expected to incur more costs in order to be successful in their subjects. However, the two groups did not differ in their general expectancies of successfully graduating. It seems that STEM students are aware of the costs and efforts which they need to invest in order to succeed in STEM. However, this awareness does not prevent them from choosing a STEM subject. At first glance, this appears to be in contrast to previous suggestions that perceived barriers might discourage students from deciding for a STEM subject, insofar as students seem to be aware of the effort but nevertheless see potential gains and advantages, which can be interpreted as a rational decision (Wang and Degol, 2013). On the other hand, there might be a relation of costs and perceived values based on cognitive dissonance theory: As students expect greater costs, they need to value the subject more.

In sum, these results point in the direction that STEM students’ need for autonomy could be satisfied to a lesser degree compared to non-STEM students. Nevertheless, STEM students value their subject more. This partly surprising pattern should be addressed in future studies which scrutinize the relation of autonomy and satisfaction with a choice.

Differences between STEM and non-STEM students also emerged regarding competence-related variables, but the pattern was not consistent. Competence refers to the perception of capability and effectiveness (Ryan and Deci, 2014). In this regard, non-STEM students, for instance, more frequently endorsed the statement that they chose their subjects based on their talents than did STEM students. In addition, non-STEM students showed higher self-esteem ratings than did STEM students. Both of these findings indicate that non-STEM students tend to attribute more competence to themselves than do STEM students. On the other hand, STEM students were more likely to report having selected their study subjects because they predicted that they would be able to earn a lot of money after graduation and because they wished to achieve a top management position, while non-STEM students were more likely to strive for middle management positions. This could be attributed as high competence perceptions in STEM students. Whether these mixed results allow conclusions to be drawn about the academic achievements of especially STEM students, such as the findings of Jang et al. (2009), is unclear. They do, however, reflect the notion put forward by Steele and Aronson (2005) that competence has a fragile character. It is possible that while STEM students are positively disposed toward their future and their future competencies after graduation (leadership; money), they lack confidence in their current competence (self-esteem). Furthermore, the analyses did not reveal any differences between STEM and non-STEM students in terms of attribution style, self-efficacy and academic self-efficacy. This indicates that both student groups are not particularly characterized by one of these constructs.

While the current results indicate that STEM students experience different kinds of competence, and less autonomy than do non-STEM students, we were especially interested in potential gender differences which might help to explain the lack of women in STEM subjects (Shen, 2013; Dasgupta and Stout, 2014).

There were, however, no interaction effects with regard to motivations to choose the specific subject of study. Concerning motivation and interest in study subject, on the other hand, analyses interestingly show that especially female STEM students differ from the non-STEM groups in the sense that they value their study subject more. This might indicate that those students who – against their gender role – choose a study subject are all the more determined to evaluate their choice positively. This is in conflict, however, with the finding that there are no differences in the reasons for choosing the study subject.

Regarding the expected investments needed, male STEM students, in contrast to their female counterparts, expect most costs, in terms of invested effort, to succeed in their study programs, followed by female non-STEM students and male non-STEM students. However, male STEM students scored higher regarding their expected professional opportunities after graduation compared to female STEM students, while no such difference was found between female and male non-STEM students. Male STEM students in particular seem to direct their focus toward future competence after graduation. No further interaction effects emerged. The interaction effects do not give reason to assume that female STEM students, in contrast to all other groups and particularly to their female non-STEM counterparts, can be characterized by, for instance, lower competence perceptions in terms of self-efficacy or a detrimental attribution style. However, the deeper analyses of female and male STEM students revealed several gender differences, which discriminate female STEM students at least from male STEM students.

Male STEM students were more likely to choose their study programs because they knew what they wanted to do after graduation, they saw a lot of professional opportunities after graduation and were more confident that they would be able to make a lot of money after graduation. Furthermore, compared to female STEM students, they were more likely to wish to work in top management. These results indicate more intense (future) competence perceptions compared to those of female STEM students, which are particularly directed at the future after graduation. Only one finding clearly showed that male STEM students currently perceive more competence than do female STEM students: The males showed higher self-efficacy scores, which is in line with previous findings (e.g., Zeldin et al., 2008). One construct which may contribute to the perception of competence is how people attribute their successes and failures (Ryan and Deci, 2014). For instance, attributing success to one’s own ability could be beneficial, while attributing the same result to luck would be less beneficial. In line with previous research (Burgner and Hewstone, 1993), we found that women in STEM score higher on controlled orientation and impersonal orientation, but that there is no difference in autonomy orientation. In sum, this indicates that while women do not necessarily perceive less competence and autonomy, and women and men attribute the results of their behavior equally to internal sources, women are also more likely to attribute results to external sources and to have a greater feeling of powerlessness regarding the results of their behavior, as reflected in their lower values for self-efficacy. In contrast to previous findings (MacPhee et al., 2013), our analysis did not reveal any differences regarding perceived academic self-efficacy or self-esteem. Although MacPhee et al. (2013) reported a gender difference in perceived academic skills, they acknowledged that by the time of graduation, women had reached equal levels of academic self-efficacy to those of men. Given that we surveyed Master’s students, it is reasonable to assume that female STEM students had already reached this point of equality.

Still, in sum there are several distinct differences between men and women regarding perceived competence and study motivation. How can this be traced back to the influence of stereotypes as discussed above? While stereotype research would not predict a STEM subject for women, it would do so all the more for men (e.g., Carli et al., 2016). Socialization in such beliefs could motivate males to choose a STEM study program, and render them confident in experiencing competence after graduation. Female STEM students, in contrast, are partly lacking the perception of competence. In line with previous stereotype research, this may also have been induced by stereotypical beliefs about women and STEM, with female STEM students assuming that their own effort, capabilities and abilities might be worth less compared to male STEM students, because STEM is a male domain (e.g., Hand et al., 2017). However, the assumption of links between stereotypical beliefs and the present findings also leads to our limitations.

### Limitations

We did not capture participants’ stereotypical beliefs about women and men. Such a consideration might have provided valuable insights into the degree to which participants’ behavior and perceptions may have been affected by these social norms. Future studies should address this issue. Moreover, we captured participants’ belonging to the binary (biological) category of sex, but used the term “gender” within this paper, due to the association with stereotypical beliefs which refer to social identity (social gender, Bem, 1993).

In addition, it would be useful to focus on social identity in order to examine whether differences in students’ perceptions could be better predicted by a more female or male identity instead of a fixed binary category. It could be possible that women in STEM and men in non-STEM have slightly different gender identities, because they decided for gender-incongruent subjects. However, we found numerous differences with regard to the reasons why people selected their majors. Although we suggest that these additional ad hoc items yielded valuable results, ratings were mostly given on the lower parts of the scales, indicating that the chosen reasons were only relevant for a minority of students. Therefore, future studies should also capture additional reasons beyond classical motivations and interest items. Moreover, some of the scales we used, especially the causality orientation scale, obtained rather low internal consistency. In the case of the causality scale we have used a reduced set of the scale’s original number of vignettes by Deci and Ryan (1985) to reduce the length of the survey. This lower number could be one reason for the low consistency (Cortina, 1993; Peterson, 1994). Nevertheless, using the full set of vignettes could be one way to increase the quality of the scale; another way could be to use scales which do not refer to vignettes.

In sum, results need to be considered with caution. As we have a comparatively large sample, we obtain significant differences which might not always be associated with high effect sizes. Therefore, implications for everyday life should only be derived from those results which also have high or at least medium effect sizes.

Furthermore, we gathered data of students from one university; interpretations might be exclusive to this particular group of students. To provide further evidence, future studies should be conducted with a student sample stemming from various universities.

## Conclusion

The bigger picture shows that there are some differences between STEMand non-STEM students in terms of their competence and autonomy perceptions, which are in favor of non-STEM students. One major contribution of the paper is the finding that STEM students seem to direct their competence perceptions toward future competence, such as achieving a position in top management, seeing numerous professional opportunities or earning large amounts of money. This also holds true for the interaction of subject and gender, revealing that men in STEM score highest, followed by their female counterparts, while this future-related competence does not seem to be important to non-STEM students (either female or male). Furthermore, male and female STEM students do not differ in their autonomous behavior. Therefore, it could be expected that both women and men will achieve their academic goals and might become professionally satisfied (e.g., Ng et al., 2006). However, there are some differences in perceived competence ratings, such as self-efficacy and leadership aspirations, which is in line with previous research (e.g., Pajares and Miller, 1994; Amon, 2017). In conclusion, it seems that female STEM students are equally autonomy-oriented as male STEM students, but have lower perceptions of their own competence, especially regarding the expectation of future success.

## Ethics Statement

This study was neither approved nor rejected by an ethical committee of the University due to lack of Central Ethics Commission in the University. However, the study idea and questionnaire were read, revised, and approved by different boards of the university: University board of the University of Duisburg-Essen, [Prof. Radke (rector); Prof. van Ackeren (vice-rector); Prof. Dr. Spitzley (vice-rector), Prof. Ziegler (former vice-rector), the (former) vice-rector for Diversity and Equality (Prof. Ziegler), the Equality Committee (e.g., former Prof. Krämer; former Dr. Mense, in total including members of all status groups of the University] and the University’s data protection officer (Dr. Lose) who ensured anonymity of participants and data protection. We hope that the inclusion and approval of all status groups of the University serve as a sufficient approval that participants of the current survey study were treated with respect and did not face unethical conduct. We conducted the study in all conscience.

## Author Contributions

SS and NK contributed to conception and design of the study. SS organized the database, performed the statistical analysis, and wrote the manuscript. Both authors read, revised, and approved the submitted version.

## Conflict of Interest Statement

The authors declare that the research was conducted in the absence of any commercial or financial relationships that could be construed as a potential conflict of interest.
